# Editors' Pick: Of Horses and Genes'

**DOI:** 10.1186/2041-2223-3-4

**Published:** 2012-02-17

**Authors:** Manfred Kayser

**Affiliations:** 1 Department of Forensic Molecular Biology, Erasmus MC University Medical Centre Rotterdam, PO Box 2040, 3000 CA Rotterdam, The Netherlands

## 

"Diamonds are a girl's best friend", and for some people horses are too. Horses hold an important place in many human societies with a role that has changed considerably over time from beast of burden and meat (and later milk) source, via transportation means, to leisure activities and even financial speculations today. It is therefore no wonder that horses also attract the interest of investigative geneticists. The recent literature provides new insights into the genetic history of horse domestication. In an article published last month, Achilli *et al*. [1] studied the diversity of maternally-inherited complete mitochondrial genomes of modern horses from Asia, Europe, the Middle East, and the Americas. Most of the 17 major haplogroups they classified were distributed over different geographic areas and were all found in modern horses from Asia. These findings, in line with earlier published work [2], indicate that multiple female horse lines were involved in horse domestication in the Eurasian steppes at (as suggested by archaeological and molecular dating) 5-7 thousand years ago (kya) [2]. Strikingly, such considerable mitochondrial DNA diversity contrasts sharply with the virtual absence of paternally inherited Y-chromosome sequence diversity in modern horses, implying that all modern horses trace back to a single male line despite their multiple maternal origins [3]. Is this the consequence of a strong sex bias during horse domestication, or was the Y-chromosome diversity already reduced in pre-domestic horses? Lippold *et al*. [4] appear to have answered this question via ancient DNA analysis of wild horse remains from Siberia and North America dated 47 to 16 kya, and one 2.8 kya domestic stallion. They demonstrated considerable ancestral Y-chromosome diversity, and concluded that the lack of paternally inherited genetic diversity in modern horses can be explained as a direct consequence of the domestication process itself. Another paper published last month [5] investigated the genetic origin and history of speed in the Thoroughbred racehorse, the most valuable breed of horse in the world. Earlier studies had shown that a C/T single nucleotide polymorphism in an intron of the MSTN gene influences the speed in the Thoroughbred [6] (although other genes should contribute as well). Bower *et al*. [5] have now demonstrated that the T-allele was ancestral and there was a single introduction of the C-allele at the foundation stage of the Thoroughbred. They also showed that in recent times the C-allele has increased in frequency in the Thoroughbred through the process of selective breeding. Another aspect of human excitement about horses, namely coat colour, was genetically investigated in a study published at the end of last year. Pruvost *et al*. [7] performed ancient DNA analysis on coat colour genes in remains from pre-domestic horses dated at Pleistocene, Mesolithic-Neolithic and Copper age times from Eurasia. They identified bay and black coat colour, and also found an allele associated with the leopard spotting pattern; notably, all these colour types were seen with horses on Paleolithic cave paintings. Thus, they concluded that Paleolithic cave paintings reflect more of the natural environment and less of the artist's imagination or spiritual stage than often assumed (at least when it comes to horses).
